# Role of trazodone in treatment of major depressive disorder: an update

**DOI:** 10.1186/s12991-023-00465-y

**Published:** 2023-09-02

**Authors:** Andrea Fagiolini, Ana González-Pinto, Kamilla Woznica Miskowiak, Pedro Morgado, Allan H. Young, Eduard Vieta

**Affiliations:** 1 Department of Molecular and Developmental Medicine, Division of Psychiatry, University of Siena School of Medicine, Viale Bracci 12, 53100 Siena, Italy; 2 Bioaraba Research Institute, Department of Psychiatry, Araba University Hospital, 01004 Vitoria, Spain; 3 CIBERSAM. University of the Basque Country, Vitoria, Spain; 4 Psychiatric Centre, Copenhagen Affective Disorder Research Centre (CADIC), Rigshospitalet, Copenhagen, Denmark; 5 Copenhagen University Hospital, Rigshospitalet, Copenhagen, Denmark; 6 Department of Psychology, University of Copenhagen, Copenhagen, Denmark; 7 Life and Health Sciences Research Institute (ICVS), School of Medicine, University of Minho, 4710-057 Braga, Portugal; 8 ICVS/3B’s, PT Government Associate Laboratory, 4710-057 Braga/Guimarães, Portugal; 9 Clinical Academic Center-Braga (2CA), 4710-243 Braga, Portugal; 10 Department of Psychological Medicine, Institute of Psychiatry, Psychology and Neuroscience (IoPPN), King’s College London Strand, London, UK; 11 South London and Maudsley NHS Foundation Trust, Bethlem Royal Hospital, Kent, UK; 12 Bipolar and Depressive Disorders Unit, Hospital Clinic, University of Barcelona, IDIBAPS, CIBERSAM, Barcelona, Spain

**Keywords:** Major depressive disorder, Trazodone, Agitation, Anxiety, Insomnia

## Abstract

Major depressive disorder (MDD) is the most common mood disorder and a leading cause of disability worldwide. Trazodone, a triazolopyridine serotonin receptor antagonist and reuptake inhibitor (SARI) antidepressant approved for major depressive disorder (MDD) in adults, has established efficacy that is comparable to other available antidepressants, and is effective for a range of depression symptoms, including insomnia, which is one of the most common and bothersome symptoms of depression. Also, trazodone’s pharmacodynamic properties allow it to avoid the side effects of insomnia, anxiety and sexual dysfunction often associated with selective serotonin reuptake inhibitor antidepressants. In this narrative review, we have summarized recent clinical trials and real-world data on trazodone, including the recently introduced once-daily formulation, which has single dose pharmacokinetic properties that maintain effective blood trazodone levels for 24 h, while avoiding concentration peaks associated with side effects. This, combined with a low incidence of weight gain, and sexual dysfunction, may improve adherence to treatment. The most common adverse effects of trazodone are somnolence, headache, dizziness and xerostomia. It has minimal anticholinergic activity but may be associated infrequently with orthostatic hypotension (especially in patients with cardiovascular disease or older adults), QT interval prolongation, cardiac arrhythmias, and rare episodes of priapism. The low liability for activating side effects, the efficacy on symptoms such as insomnia and psychomotor agitation and the rapid onset of action make it useful for many depressed patients, both in monotherapy at nominal dosages of 150–300 mg/day, and in combination with other antidepressants at lower dosages.

**Supplementary Information:**

The online version contains supplementary material available at 10.1186/s12991-023-00465-y.

## Introduction

Major depressive disorder (MDD) is the most common mood disorder and a leading cause of disability worldwide [1], affecting multiple functional domains that impact education, work productivity, and quality of life (QoL). Treatment can include psychotherapy, psychopharmacotherapy, or combinations of these. Antidepressant therapy, with the goal of achieving remission of symptoms and recovery of function is the most common treatment for severe depression [2, 3]; however, these goals are achieved in only about half of patients, and many experience relapses before recovery is achieved. This low success rate may be due to a lack of adequate treatment [4], or low treatment adherence [5]. Patients who require a longer treatment duration to achieve remission are at higher risk of early relapse [6]. Patients who fail to achieve remission after adequate trials with two or more different antidepressants have been described as *treatment-resistant* [7]. *Difficult-to-treat depression* (DTD) is a related concept describing “depression that continues to cause significant burden despite usual treatment efforts [8–10].

This narrative review provides an update on recent clinical data for trazodone in MDD and its place in current treatment strategies for MDD, with a focus on the once-daily trazodone Contramid^®^ formulation, and symptoms that do not respond well to other treatments. In preparing it, we have conducted a literature search in PubMed using the keywords “trazodone” and “major depressive disorder” and focused on updating the 2012 review published by Fagiolini et al. [11].

## Trazodone

Trazodone is a triazolopyridine derivative that inhibits the reuptake of serotonin and blocks histamine and alpha-1-adrenergic receptors. It is marketed in a variety of forms, including a once-daily formulation. Clinical studies have shown that the anti-depressive efficacy of trazodone, when administered at ≥ 150 mg/day, is comparable to that of tricyclic antidepressants [12–14], selective serotonin reuptake inhibitors [15–18], and serotonin-norepinephrine receptor inhibitors [19], (reviewed in [11]). Trazodone is generally well tolerated and may have additional advantages for patients who have both major depression and prevalent sleep disturbances [11].

### Pharmacokinetic properties of trazodone formulations

**Fig. 1:**
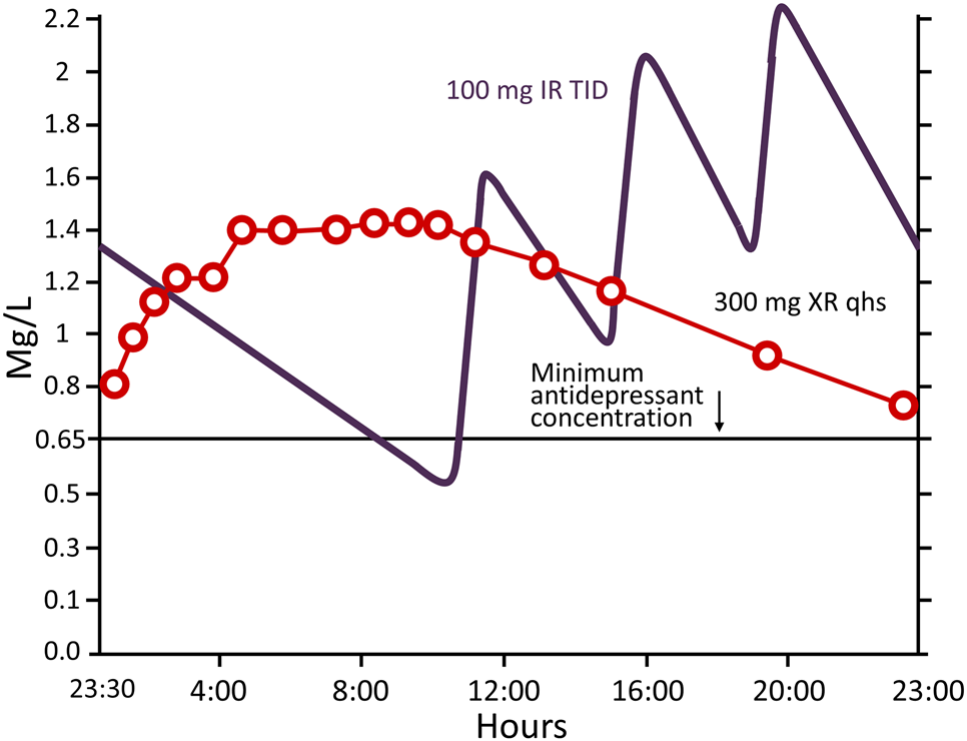
Pharmacokinetic properties of trazodone IR (immediate-release formulation, purple) dosed at 100 mg three times per day, and trazodone XR (extended-release, red) dosed at 300 mg once daily at night, showing plasma drug levels with respect to the minimum antidepressant concentration of 0.65 Mg/L over a 24 h period. (From Stahl [22])

The immediate-release formulation may be used as augmentation with another treatment, especially in patients who have major depressive disorder and accompanying symptoms such as insomnia, anxiety, irritability, or mild psychomotor agitation; however, its use requires administration in three separate doses, unless the medication is prescribed at relatively low doses, for example to target the symptom of insomnia. The immediate-release formulation is associated with fluctuations in plasma drug concentrations that increase the likelihood of adverse effects [11]. The Contramid^®^ formulation is administered once daily at doses of up 300 mg at night, and provides controlled release of trazodone over 24 h [20]. Administration of trazodone Contramid^®^ once daily for 7 days results in plasma AUC that is similar to that of the immediate-release formulation administered three times daily, but with peak maximum concentration (C_max_) that are 43% lower [21] **(**Fig. 1**)**.

This improves tolerability while increasing the probability of maintaining drug concentrations at levels adequate for antidepressant effectiveness.

### Pharmacodynamic properties of trazodone and relationship to clinical efficacy

#### Dose-dependence of activity

**Fig. 2:**
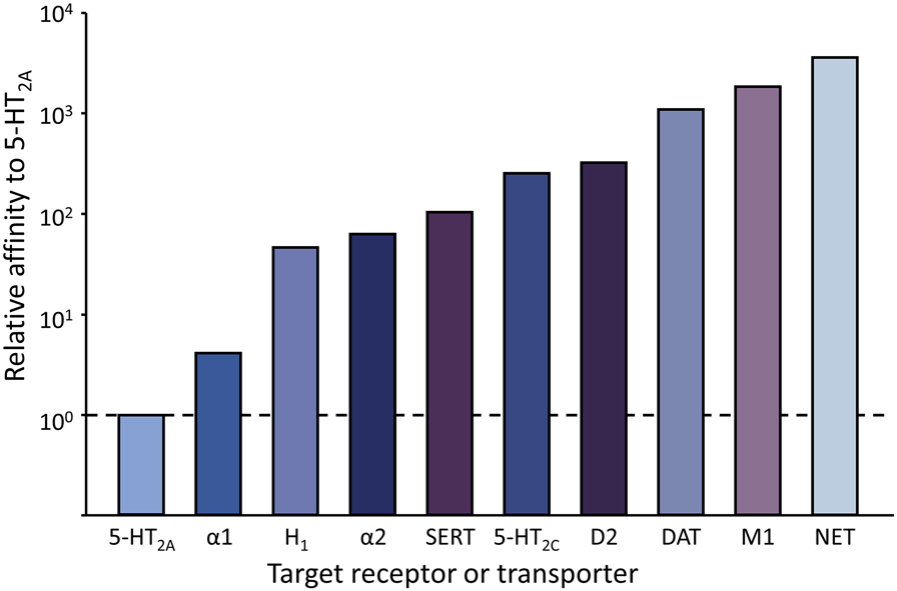
Trazodone affinities at neurotransmitter receptors and transporters. The most potent action is antagonism at 5-HT_2A_ receptors. 5-HT_2A_ and 5-HT_2C_, subtypes of 5-hydroxytryptamine receptors; α1 and α2, subtypes of alpha adrenergic receptors; H1, histamine 1 receptor; SERT, serotonin reuptake transporter; D2, dopamine 2 receptor; DAT, dopamine transporter; M1, muscarinic 1 cholinergic receptor; NET, norepinephrine transporter. (From Stahl [22]

Trazodone is a dose-dependent multifunctional drug that acts only on its highest affinity binding sites at low doses but has additional pharmacological actions at higher doses (Fig. 2).

Doses of 150–600 mg are required to saturate serotonin transporters and achieve antidepressant activity (Box 1). At this dose, trazodone is a multifunctional serotonergic agent that inhibits the serotonin transporter (SERT) and antagonizes 5-HT2A and 5-HT2C receptors [22, 23], making it a multifunctional SARI.

**Box 1:** Summary of pharmacodynamic properties at 150–600 mg/day

• Antidepressant properties–antagonism of 5-HT2A and 5-HT2C receptors; inhibition of the serotonin transporter; 5HT1A partial agonism
• Anxiolytic effects—5HT2c antagonism
• Sleep improvement—5HT2a antagonism (H1 antagonism) and α1 receptor antagonism
• Effects on agitation—α1 blockade and 5HT2a antagonism
• Minimal anticholinergic effects

Trazodone doses between 25 and 150 mg do not achieve antidepressant activity but have hypnotic activity through antagonism of 5-HT_2a_, H_1_, and α1 receptors. Estimates of brain receptor occupancy based on a pharmacokinetic model support the multimodal activity of trazodone, which may contribute to the rapid onset of its antidepressant action and its effectiveness for a variety of symptoms in depressed patients [24].

#### Rapid onset of action

Generally, the effectiveness of antidepressants remains suboptimal, and part of this may be due to their delayed onset of action. However, trazodone appears to have a rapid onset as seen in results from controlled clinical trials [25, 26], and the real-world setting [27]. This rapid onset of action may result from the efficacy on depression symptoms such as insomnia, anxiety, irritability, and psychomotor agitation [11, 28], as well as from the combined effects of partial 5-HT1A receptor agonism and serotonin transporter inhibition [29].

### Safety and tolerability

Trazodone is generally well tolerated in patients with MDD and, compared to SSRIs and SNRIs it has a low risk for anxiety, insomnia, and sexual dysfunction, due to its simultaneous inhibition of 5-HT2A and 5-HT2C receptors, and serotonin transporters, along with its anti-alpha adrenergic and anti-histaminergic properties [11, 30]. The most common side effects include somnolence, headache, dizziness, and xerostomia.

Efforts should be made to minimize the impact of these side effects. Bedtime administration can reduce the impact of somnolence. Xerostomia, depending on severity, can cause difficulty in swallowing and increase the risk of oral infections and tooth decay, and may negatively affect quality of life with consequences for treatment adherence. Patients should be instructed to report side effects, including xerostomia, to the clinician, who may suggest strategies for managing them.

#### Other side effects

- Arrhythmias—An ECG trial identified a *modest*, dose‐dependent effect of trazodone on cardiac repolarization, suggesting that caution is warranted in patients with concomitant medications known to prolong the QT interval [31].
- Orthostatic hypotension, secondary to α1-adrenergic antagonism, may occur in patients receiving trazodone and could be more severe in patients also receiving antihypertensive therapy, especially in older adults or patients with heart disease [11].
- Priapism is a rare AE with trazodone that is likely due to α-adrenergic antagonism. To reduce the risk, trazodone should be used with caution in patients with anatomical penis deformation or conditions including multiple myeloma or sickle cell anemia.

Tolerability of trazodone is further improved with the extended-release Contramid^®^ formulation, which avoids concentration peaks associated with orthostatic hypotension and sedation, while allowing treatment to be initiated at a dose that is effective for depression [28]. In the RCT by Sheehan et al. side effects compared to placebo were mostly mild and their incidence decreased over time [25].

### Pregnancy and lactation.

Trazodone is classified in the US FDA pregnancy category C; however, preliminary data suggest that maternal exposure to trazodone in early pregnancy is not associated with adverse outcomes [32]. Limited information indicates that trazodone levels in milk are low and would not be expected to cause any adverse effects in breastfed infants, especially if the infant is older than 2 months or when doses of 100 mg or less are used at bedtime for insomnia. Based on a safety scoring system for the use of psychotropic drugs during lactation, use of trazodone is considered “possible with caution” when necessary [33].

### Update on clinical trials of trazodone in MDD

Since the last major review [11], three prospective trials have investigated the efficacy and safety of trazodone in patients with MDD [26, 34, 35].

The efficacy and safety of once-daily trazodone (Contramid^®^ formulation) was compared to that of extended-release venlafaxine XR in an 8-week active controlled study that randomized 324 adults with MDD to receive trazodone 300 mg/day (n = 166) or venlafaxine 75–225 mg/day (n = 158) [26]. The primary efficacy endpoint was non-inferiority of the mean change from baseline on HAM-D-17 at the final study visit on day-56 in the ITT and per protocol populations. Secondary endpoints included the mean change from baseline in the Montgomery-Asberg Depression Rating Scale (MADRS) score at the final visit; (ii) Clinical Global Impression Severity (CGI-S) and Improvement (CGI-I) results at day-56, and the rates of response (≥ 50% reduction from baseline in HAM-D-17 total score) and remission (HAM-D-17 score ≤ 7) at the final visit. Patients were monitored at weeks 1, 3, 5, and 8, with additional safety visits after dose escalations.

The mean (SD) treatment doses administered were 311.4 ± 48.7 mg/day for trazodone and 84.1 ± 29.9 mg/day for venlafaxine. Both treatments effectively reduced HAM-D-17 scores at week 8 compared to baseline in the ITT population (trazodone − 12.9 ± 6.82, venlafaxine − 14.7 ± 6.56), and the per-protocol population (trazodone [n = 122] − 15.4 ± 5.32, venlafaxine [n = 127] − 16.4 ± 5.39). Efficacy was significantly higher in the venlafaxine group, while the reduction in HAM-D-17 score was significantly greater at day 7 in the trazodone group, compared to the venlafaxine group (*p* < 0.05). At treatment day 56, a response was achieved by 65.4% of patient in the trazodone group and 76.3% in the venlafaxine group (p < 0.05), while clinical remission was ongoing in 37.7% of patients in the trazodone group and 52.0% in the venlafaxine group.

Most reported AEs were mild or moderate in severity and a similar percentage of patients had treatment-related adverse events. The most frequent AEs in the trazodone group were dizziness and somnolence, while in the venlafaxine group they were nausea and headache [26].

Treatment-resistant depression [7] and difficult-to-treat depression [9] are common and burdensome. Effective treatment should address symptoms known to compromise response, functionality, and/or quality of life [8]. Pharmacological augmentation may be an effective component of a comprehensive, measurement-based strategy. Fang et al. assessed the effectiveness and tolerability of 5 different augmentation partners to pair with paroxetine 20 mg/day in an 8-week randomized study of 225 adults with treatment-resistant depression (TRD) [34]. Trazodone 100 mg/day was among the treatments assessed, along with buspirone 30 mg/day, risperidone 2 mg/day, thyroid hormone 80 mg/day, and valproic acid 600 mg/day. There were no statistically significant differences among groups for the primary outcome of remission defined as HAM-D-17 score of ≤ 7 at study end. Tolerability was also similar among groups, and no serious adverse events were reported during the study period. In the paroxetine plus trazodone group (n = 47), 20 patients (43%) achieved remission and 29 patients (62%) responded with a ≥ 50% reduction in HAM-D-17 total score at study end. There was no placebo group for comparison.

Zhang et al. assessed the efficacy, safety, and clinical benefit of prolonged-release trazodone (Trittico^®^; also referred to as slow-, delayed-, or controlled-release), which was administered twice daily in a 6-week randomized placebo-controlled flexible-dose study that randomly assigned 363 adults with MDD to receive prolonged release trazodone (150–450 mg/day) or placebo [35]. The prolonged-release trazodone group had received a mean maximum trazodone dosage of 273 mg/day, and had a significantly greater improvement from baseline in mean HAM-D-17 total score at study end (primary outcome) compared to placebo (− 11.07 vs. − 8.29, *p* < 0.001).

The rate of response defined as a ≥ 50% improvement in HAM-D-17 total score, was 59.6% (109/183) with prolonged-release trazodone vs. 37.2% (67/180) with placebo (*p* < 0.001), and the remission rate defined as a HAM-D-17 total score ≤ 7, was significantly higher with prolonged-release trazodone (35.5% [65/183] vs. 22.2% [40/180]; *p* = 0.005). Consistent with previous studies [25], sleep quality, assessed using the Pittsburgh Sleep Quality Index, was significantly improved from baseline with prolonged-release trazodone compared to placebo (*p* < 0.001).

Trazodone was well tolerated. AEs (n = 241) were mostly mild or moderate, and were reported by 156 of the 366 patients, including 100 patients who received prolonged-released trazodone (54.1%) and 56 patients who received placebo (30.9%). Dizziness, xerostomia, and somnolence were the most common; dizziness and somnolence were significantly more common with trazodone.

### Recent real-world data

Several recent real-world studies have investigated the effectiveness and tolerability of trazodone [27, 36, 37]. Miljevic et al. conducted an eight-week multi-center open-label observational study to evaluate the effectiveness and tolerability of prolonged-release trazodone, in this case administered as a single daily dose in the evening to 242 adults with MDD experiencing a depressive episode without psychotic features [36]. Trazodone monotherapy was initiated at 50 mg and titrated to 150 mg over 2 weeks, with the possibility of increasing the dose up to 300 mg/day based on response (HAM-D-17) at week 2 compared to baseline. The 300 mg dose was administered to 54 patients. Outcomes were assessed at weeks 2, 4, and 8, using the HAM-D-17, HAM-A-14, and the CGI-S, and response was defined as ≥ 50% improvement from baseline on the HAM-D-17 or HAM-A-14. Significant improvements in depression were apparent at the first follow-up visit, with the HAM-D-17 baseline value of 23.74 (95% CI 23.15 to 24.33) reaching 17.32 (95% CI 16.59 to 18.06) after 2 weeks of treatment (*p* < 0.001) and continuing to improve to 8.67 (95% CI 8.02 to 9.32) at week 8 (*p* < 0.001). Similar improvements were observed on the HAM-A-14 scale. Responses were achieved by 12% of patients at week 2 and by more than 80% had responded at study end.

Adverse events (n = 73) were experienced by 52 patients, were mostly mild (62%), and were reported mainly at the first follow-up visit, when 42 patients reported events. The most frequent side effects were headache, sedation, and nausea. Severe side effects (vertigo or nausea) were reported by 11 patients. Overall, the results were similar to those from clinical trials [11], and supported the effectiveness and tolerability of trazodone for treating depression in routine clinical practice.

Some antidepressants have an initial delay before achieving a therapeutic response [38]. Intravenous administration has been suggested to accelerate attainment of steady-state therapeutic blood levels and treatment effects [39, 40]. A real-world study compared intravenous administration followed by oral dosing of trazodone vs clomipramine in 42 outpatient adults with MDD who were experiencing a major depressive episode [37]. Treatment was selected based on clinical judgement, with 26 patients receiving trazodone and 16 receiving clomipramine. Intravenous trazodone (25–100 mg/day, mean 44.2 ± 11.0 mg) or clomipramine (25–75 mg/day, mean 29.7 ± 9.30 mg) was administered for 1 week, with dosages based on the severity of depressive symptoms. Patients then switched to oral extended-release (Contramid^®^) trazodone (150–300 mg/day, mean 161.5 ± 67.4 mg) or clomipramine (50–225 mg/day, mean 98.4 ± 59.9 mg) for 4 weeks.

Patients were assessed at baseline and again after 1, 2, and 6 weeks by blinded raters using the HAM-D-17 and other psychometric scales. Response was defined as a ≥ 50% reduction in the HAM-D-17 total score and remission as achieving a HAM-D-17 total score < 8. The response rate at study end was 46.1% (12/26) in the trazodone group and 18.8% (3/16) in the clomipramine group. Remission was achieved by 34.6% (9/26) of patients receiving trazodone and 12.5% (2/16) of patients receiving clomipramine.

Side effects were reported by 4/26 patients receiving trazodone (2 sedation, 1 rash, and 1 dizziness), and 9/16 patients who received clomipramine (4 xerostomia, 1 sedation, 1 headache, and 1 dizziness). None of the patients discontinued treatment because of adverse events.

Češková et al. conducted a prospective real-world study to evaluate the efficacy, tolerability, and safety of once-daily trazodone Contramid^®^ in 85 adults with moderate to severe MDD in routine clinical practice [27]. Trazodone Contramid^®^ was effective in previously treated and treatment naïve patients, including those with depressive episodes not responding to previous antidepressant treatment. Mean reductions from baseline in overall MADRS score were significant after 1 week of titration (27.4 to 21.2; *p* < 0.001) decreased further after 4 weeks of full dosage treatment (7.9; *p* < 0.001), when scores for all MADRS items were significantly lower than baseline values. Depression severity also decreased, with 71/80 patients (94%) achieving a CGI-S of ≤ 3 after 5 weeks. Follow-up visits revealed that 79% of patients were still receiving trazodone at week 9 and 71% at week 21, and investigator-assessed depression status compared to the previous visit was either unchanged or improved in all patients. Investigator-reported treatment tolerability was good or excellent for all patients; the most frequent drug-related AEs were somnolence and fatigue.

Siwek et al. conducted a 12-week open-label study to compare the effectiveness of trazodone Contramid^®^ vs. SSRIs for adults with MDD (n = 76) [41]. Based on clinical presentation, clinicians assigned 42 patients to trazodone (mean dose 209.4 mg/day), and 34 patients to an SSRI (25 sertraline and 9 escitalopram; mean dose 21.7 fluoxetine equivalent mg/day).

Assessments at baseline and weeks 2, 4, 8, and 12 included the change in depression severity (MADRS, Quick Inventory of Depressive Symptomatology [QIDS]), with response corresponding to a ≥ 50% reduction on the MADRS or QIDS, or improvement on the CGI-I. Response rates achieved after 12 weeks were similar in both groups, but trazodone was more effective than SSRIs at reducing insomnia severity on the Athens Insomnia Scale (− 12.5, 95% CI − 15.4 to − 9.5 vs. − 3.7, 95% CI − 5.7 to − 1.8; *p* < 0.001). Both treatments were well-tolerated; one patient discontinued due to adverse effects in each group (somnolence with trazodone, and hypomania with sertraline).

### Candidate situations and populations for trazodone

MDD is heterogeneous [42, 43], and involves a variety of symptoms that may respond variably to a given treatment. For examples of clinical applications see Additional file 1. Because of its unique mechanism of action and low risk of adverse anticholinergic effects and sexual side effects, trazodone has several advantages when addressing comorbid anxiety, agitation, or insomnia [11], and has been used off label, especially for treating insomnia and anxiety [44]

Several studies have compared the effectiveness of trazodone in treating depression to other classes of antidepressants, including selective serotonin reuptake inhibitors (SSRIs) such as fluoxetine, paroxetine, sertraline, citalopram, and escitalopram, serotonin-norepinephrine reuptake inhibitors (SNRIs) like venlafaxine and mirtazapine, inhibitors of norepinephrine and dopamine reuptake (bupropion), and tricyclic antidepressants (specifically amitriptyline and imipramine) [11]. The consistent findings of these studies indicate that trazodone's efficacy is comparable to that of these other antidepressants. However, trazodone's mechanism of action makes it well-suited for addressing certain aspects of depression, including insomnia, agitation, anxiety, and irritability. It sets itself apart from benzodiazepines due to its lower risk of abuse, dependence, and tolerance. In contrast to tricyclic antidepressants, it typically avoids causing anticholinergic side effects. Unlike mirtazapine, it generally does not lead to increased appetite or body weight. Moreover, unlike certain selective serotonin reuptake inhibitors, it does not impact the levels of other drugs in the bloodstream through interactions with cytochrome P450 enzymes. Nonetheless, trazodone is not without potential drawbacks, and special caution should be exercised in patients with an elevated risk of arrhythmias or falls due to excessive sedation or orthostatic hypotension. The utility of the available trazodone formulations in different clinical settings are summarized in Box 2.

**Box 2:** Different forms of trazodone present options to tailor treatment based on individual patient symptoms and requirements

• Oral immediate-release (IR) seems well-suited for treating mild to moderate major depression in patients with initial insomnia or periods of agitation or irritability during specific times of the day, as it can achieve higher blood concentration peaks at those times [45]
• Slow release (SR, sometimes called delayed or controlled release) may meet the needs of patients with mild to moderate major depression, both initial and central insomnia, and moderate to severe daytime or nighttime anxiety. It results in blood concentration peaks that are lower but sustained compared to the IR form, and higher but more rapidly declining peaks than the once-daily ER formulation [46]
• Once a day extended release (ER, also known as XR or COAD) ensures a consistent release of trazodone over 24 h and is appropriate for managing major depression accompanied by early, central, or late insomnia, as well as daytime or nocturnal anxiety, including severe depression requiring continuous maintenance of therapeutic blood levels [20, 21]
• Intramuscular-intravenous (IM-IV) solutions might be beneficial for patients who cannot take oral medications [47]

### Residual insomnia in treated MDD

Insomnia is a common symptom in MDD [48] and is associated with substantial clinical and economic burden [49, 50]. Insomnia may be exacerbated by the activating effects of some antidepressants [51]. Trazodone has hypnotic action at doses lower than those used for treating depression (i.e., < 150 mg/day), which is primarily due to its ability to block 5-HT2A, H1, and alpha-1 adrenergic receptors [22]. The results of a systematic review suggest that trazodone is effective for insomnia, although its use for this indication is off label [52].

Results from RCTs have shown beneficial effects on sleep disorders with trazodone in the immediate-release [19, 53] and prolonged-release formulations [17, 18]. A post-hoc analysis [54] of the placebo-controlled study on once-daily extended-release trazodone (Contramid^®^) by Sheehan et al. [25], revealed that patients with MDD who had been randomized to receive trazodone had significant improvements in insomnia-related symptoms, and had the greatest improvement among the HAM-D-17 and MADRS items. This analysis also confirmed that the antidepressant effect of trazodone is independent of its effects on insomnia. In a double-blind RCT comparing trazodone Contramid^®^ with the active control venlafaxine XR in patients with MDD, both treatments significantly reduced HAM-D-17 total scores at day 56, while trazodone was significantly more effective at reducing the HAM-D-17 items related to insomnia at all time points [26].

### Major depressive episodes with mixed features

Concomitant manic symptoms not meeting bipolar disorder criteria occur during major depressive episodes in approximately one in five patients with major depressive disorder [55]. Mixed features correlate with more severe disease, longer time to recovery, and poor response to treatment [56]. This condition is recognized in the 5th edition of the American Psychiatric Association diagnostic and statistical manual of mental disorders, where it is described as major depressive episodes with mixed features (MDE-MF), and defined as a major depressive episode accompanied three or more (hypo)manic symptoms that occur on most days [57]. Anxiety and agitation are excluded from the DSM5 definition, but these so-called overlapping symptoms are often associated with mixed features in clinical practice and are included in the research‐based diagnostic criteria used in the Improving Diagnosis, Guidance and Education (BRIDGE)-II-MIX study [58, 59]. A cluster analysis of the BRIDGE Study suggested that these overlapping symptoms increase sensitivity for detecting MDE-MF [60].

Fagiolini et al. [61] proposed that patients with MDD who experience an MDE-MF, with or without psychomotor agitation (i.e., irritability, restlessness, lack of impulse control, high suicide risk, racing thoughts, increased energy, severe insomnia, or reduced need for sleep), should receive an antimanic agent during the acute episode, and be considered for treatment with an antidepressant that does not increase dopamine or norepinephrine levels, such as citalopram or trazodone. Some antidepressant treatments exacerbate agitation and worsen mania symptoms during mixed episodes, without resolving depressive symptoms. Trazodone has a low propensity for causing agitation or psychomotor activation, which allows trazodone to be used in MDD patients with isolated (hypo)manic symptoms.

### Patients with MDD and alcohol use disorder

Individually, MDD and AUD pose significant challenges to public health due to their widespread occurrences and the serious impact they have on clinical and functional well-being [1, 62]. Their co-occurrence is common, yet effective treatment strategies for addressing both conditions together remain limited [63, 64]. Symptoms of anxiety and insomnia are often observed in individuals with AUD [65, 66], and in patients with MDD [48, 67].

Trazodone improved polysomnographic sleep efficiency after detoxification in patients with AUD in a small placebo controlled study (n = 16), with an immediate effect that was sustained at 4 weeks [68]; however, a placebo-controlled trial in subjects with self-reported sleep disturbances after alcohol detoxification revealed an improvement in sleep quality while receiving low dose trazodone (50 to 150 mg/day), but an increase in alcohol intake after treatment discontinuation at week 12 [69]. Di Nicola et al. retrospectively evaluated the effect of extended-release trazodone, flexibly dosed at 150–300 mg/day, in outpatients with MDD and comorbid alcohol use disorder (n = 100) [70]. After 6 months of treatment, there had been a significant improvement in depressive symptoms (primary outcome), with 55% of patients achieving remission. Similar improvements were observed in measures of anxiety, sleep quality, functioning, quality of life, clinical global severity, as well as alcohol craving.

### Patients with history of non-adherence to antidepressants

Lack of adherence to therapy is a major contributor to poor outcomes in MDD, and simpler treatment regimens may overcome this [71]. Prescribing poorly tolerated antidepressants or agents with complex regimens can compromise patient adherence to antidepressant treatment [72]. The low incidence of side effects with trazodone and the simplified administration of once daily oral dosing at bedtime may improve tolerability and *potentially* lead to improved adherence.

## Conclusions

Trazodone is a well-established antidepressant with good tolerability and a comparable efficacy profile to other antidepressants, as demonstrated in both clinical trials and clinical practice. Trazodone may be particularly useful in patients with major depressive disorder and accompanying symptoms such as insomnia, anxiety, irritability, or psychomotor agitation.

Appropriate doses for achieving antidepressant effect in adult patients are usually 150–300 mg/day and are often higher than the doses used when trazodone is prescribed to enhance the antidepressant effect of another drug (e.g., to treat insomnia). Trazodone is usually well tolerated and carries a low risk of anticholinergic side effects (e.g., constipation, urinary retention, xerostomia), weight gain, and sexual side effects.

The Contramid^®^ ER formulation of trazodone provides sustained release over a 24-h period, avoiding peaks and troughs in drug concentration and the associated increase in occurrence or severity of certain side effects (e.g., sedation or orthostatic hypotension). This allows once daily dosing, which increases convenience and patient adherence to therapy.

### Supplementary Information

**Additional file 1:** Virtual patient cases.

## Data Availability

Data sharing is not applicable to this article as no datasets were generated or analysed during the current study.

## Abbreviations

AEs: Adverse Events
CGI-I: Clinical Global Impression Improvement
CGI-S: Clinical Global Impression Severity
DTD: Difficult-to-Treat Depression
HAM-D-17: Hamilton Rating Scale for Depression
ITT: Intention to treat
MADRS: Montgomery-Asberg Depression Rating Scale
MDD: Major Depressive Disorder
MDE-MF: Major Depressive Episodes with Mixed Features
QIDS: Quick Inventory of Depressive Symptomatology
QoL: Quality of Life
RCT: Randomized Clinical Trial
SARI: Serotonin receptor Antagonist and Reuptake Inhibitor
SERT: Serotonin Transporter
SNRIs: Serotonin and Norepinephrine Reuptake Inhibitors
SSRIs: Selective Serotonin Reuptake Inhibitors
TRD: Treatment-Resistant Depression
