# Supplementary material for: Role of trazodone in treatment of major depressive disorder: an update
Source: Ann Gen Psychiatry. 2023 Sep 2;22:32. doi: 10.1186/s12991-023-00465-y (PMC10474647; doi:10.1186/s12991-023-00465-y)
Supplement: Supplementary file 1 — Additional file 1: Virtual patient cases. [file 12991_2023_465_MOESM1_ESM.docx]

Additional File 1. Virtual patient cases

The following case examples are virtual patient cases. Although they mirror real-life clinical scenarios based on the authors’ clinical experience, they do not refer to specific patients.

Case 1: Depression with agitation and insomnia

Frank is a 41-year-old male high school graduate who is currently employed at a real estate agency where his responsibilities include showing houses to potential buyers. Frank has been married for just over a year to Martha (age 40), a legal secretary who works in a law office. They live in a rented house and have no children.

His father is 63 years old, has a high school diploma, is employed and in good health. His 62-year-old mother did not graduate from high school, has never been employed, and has a history of anxiety and depression. His 32-year-old brother is a salesman, with a long history of bipolar disorder.

On his first visit Frank reported feeling sad, lacking interest and motivation, and feeling restless, for at least one month. He also mentioned difficulty falling asleep, multiple nocturnal awakenings and inability to fall back asleep after 3:00-4:00 in the morning. He also reported feeling insecure at work, excessive worrying about his responsibilities and fixation on making mistakes. For a few weeks, moreover, he no longer wanted to eat and has lost weight. He reported occasionally thinking that life is not worth living but has never thought that it would be better to die. He mentioned having periods when he feels very agitated but denies having periods with elevation of mood or energy. Psychotic symptoms are absent.

We proposed starting trazodone Contramid® 150 mg in the evening and asked him to call us after 3-4 days and return for a visit after 1 week. At the phone interview, he reported feeling somewhat better and having noticed an improvement in sleep and reduction in anxiety but still feeling very uncertain and depressed, with difficulty making decisions and feeling overwhelmed by work responsibilities. His only side effect was s light morning sedation. We decided to increase the dose of trazodone Contramid® to 1 1/2 tablet of 150 mg (dose: 225 mg), to be taken in the evening. At his office visit three days later, Frank’s situation was unchanged; therefore, we decided to increase his dose to one 300 mg tablet and asked him to return after another week. At that point, the patient had improved greatly and reported that he has noticed an improvement in morning sedation as well. At his next visit two weeks later, his symptoms and side effects were completely resolved.

Case 2: Depression with mixed symptoms, in the absence of bipolar disorder

Elise is 39-year-old woman who is married and unemployed. Her parents are retired financial consultants. She reported a family history (maternal) of anxiety and mood disorders, not better specified. Elise is obese, with prediabetes and sleep apnea. In recent weeks, she has become progressively more anxious, and sometimes moderately agitated, with mood fluctuations, from depression to anxiety, irritability, a sense of inadequacy, binge eating crises, difficulty falling asleep with multiple awakenings throughout the night and early morning. Elise has lost interest in things she used to enjoy, such as reading, and can no longer concentrate even in front of the television. She denies having current or previous symptoms of racing thoughts, periods of mood elevation, reduced need for sleep, increased energy, or hyperactivity.

She reported having had two similar episodes in the past, both lasting more than 6 months and for which she was never treated. Trazodone Contramid® was started at 150 mg in the evening and she was scheduled for a return visit in 1 week. At the visit, she reports an improvement in sleep and anxiety but also that her mood was still depressed and interest level essentially zero. Treatment was well tolerated, except for mild headache and dizziness during the first 2-3 days, which resolved without intervention. The trazodone dose was increased to 225 mg (1 and a half tablets) per day and another visit was scheduled in 10 days. At the next visit, further improvement was observed, without significant side effects. Therefore, it was agreed to increase the Trazodone Contramid® dose to one 300 mg tablet taken in the evening. The patient returned after 2 weeks and reported feeling well and having no tolerability problems. At the next visit in 3 weeks all symptoms had resolved, and the Elise had started to lose weight, thanks to a diet that she seems to be very motivated to follow.

Case 3: Depression with generalized anxiety disorder and benzodiazepine abuse

Susan is a 55-year-old woman, divorced, without children, who has worked various jobs, including bartender, baker, caregiver, and cashier. Her symptoms began two years before the first visit, in conjunction with losing her employment, and included depressed mood, insomnia, apathy, anhedonia, difficulty managing the house, loss of interest, and increased appetite. Her symptoms had gradually worsened, with the onset of excessive anxiety and worry, accompanied by restlessness, fatigue, difficulty concentrating, irritability, muscle tension and exaggerated alarm responses. Her family doctor prescribed clonazepam, and Susan gradually increased her dosage up to about 8 mg/day. Recently, she presented with depressed mood, severe anxiety, difficulty concentrating, memory lapses, increased appetite, loss of interest, suicidal ideation, ruminations with doubts about decision-making, and ideation of guilt and failure. We decided to hospitalize her and start Trazodone Contramid ® 150 mg in the evening, tapering her clonazepam dose to 6 mg on the first day, 4 mg on the second day, 3 mg on the third and fourth days, 2 mg on the sixth and seventh days, 1 mg on days 8 and 9, followed by discontinuation. While in hospital, Susan received psychoeducation and supportive psychotherapy. After 3 days, trazodone was increased to 225 mg, and 3 days later to 300 mg. There has been a gradual and progressive improvement in mood, cognitive function, interests, anxiety, and insomnia. The improvement was partial; therefore, we prescribed an augmentation treatment with quetiapine prolonged release, a drug indicated for the adjunctive treatment (add-on) of major depressive episodes in patients with major depressive disorders that demonstrate a suboptimal response to antidepressant monotherapy. Susan started with 50 mg quetiapine prolonged release in the morning, gradually increased to 200 mg, always in the morning, while maintaining Trazodone Contramid® 300 mg in the evening. Her symptoms continued to improve, and she was discharged. During outpatient therapy, Susan continued to complain of mild sedation. Quetiapine was then gradually decreased to 50 mg in the morning, and discontinued after 6 weeks, with full resolution of symptoms and side effects. She continues Trazodone Contramid® 300 mg in the evening.

Case 4: Depression in patient with cognitive impairment

Ann is a 76-year-old widow with two children. For about 5 years she has presented symptoms that began with minor attention problems, difficulty remembering words and names for everyday objects, planning activities, abstract thinking. Her condition has gradually worsened, with memory loss, difficulty recognizing familiar people and places, temporal disorientation, inability to acquire new information, and a gradual tendency to neglect personal safety, hygiene, and nutrition. For these reasons, she now lives in an assisted care residence. In recent weeks, Ann has experienced a sharp worsening of apathy, with almost total loss of interest, refusal to leave her bed or eat, insomnia, anxiety, episodes of uncontrollable crying, depressed mood, and loss of the will to live. Although some symptoms are attributable to dementia, the clinical staff reports an evident affective involvement, suggesting a depressive episode superimposed on the cognitive impairment. The patient is being treated with memantine 10 mg/day. It was decided to start Trazodone Contramid® 150 mg (half a tablet) in the evening, which the patient tolerates well. After 3 days, insomnia improved, anxiety was reduced, and the patient resumed eating a little. The Trazodone Contramid® dose was increased to 300 mg (one tablet) and the patient had a gradual regularization of sleep, decrease in anxiety, resolution of crying episodes, further improvement of appetite and slight but progressive improvement of interests. After about 5 weeks, Ann’s condition had returned to the best level of the past 2 years.
